# Chronic Arachidonic Acid Administration Decreases Docosahexaenoic Acid- and Eicosapentaenoic Acid-Derived Metabolites in Kidneys of Aged Rats

**DOI:** 10.1371/journal.pone.0140884

**Published:** 2015-10-20

**Authors:** Masanori Katakura, Michio Hashimoto, Takayuki Inoue, Abdullah Al Mamun, Yoko Tanabe, Makoto Arita, Osamu Shido

**Affiliations:** 1 Department of Environmental Physiology, Shimane University Faculty of Medicine, Izumo, Shimane, Japan; 2 Laboratory for Metabolomics, RIKEN Center for Integrative Medical Sciences, Yokohama, Kanagawa, Japan; St. Joseph's Hospital and Medical Center, UNITED STATES

## Abstract

Arachidonic acid (ARA) metabolites produced by cyclo-oxygenase and lipoxygenase are important mediators maintaining physiological renal function. However, the effects of exogenous ARA on kidney function *in vivo* remain unknown. This study examined the effects of long-term oral ARA administration on normal renal function as well as inflammation and oxidative stress in aged rats. In addition, we measured levels of renal eicosanoids and docosanoids using liquid chromatography–tandem mass spectrometry. Control or ARA oil (240 mg/kg body weight/day) was orally administered to 21-month-old Wistar rats for 13 weeks. Levels of plasma creatinine, blood urea nitrogen, inflammatory and anti-inflammatory cytokines, reactive oxygen species, and lipid peroxidation were not significantly different between the two groups. The ARA concentration in the plasma, kidney, and liver increased in the ARA-administered group. In addition, levels of free-form ARA, prostaglandin E_2_, and 12- and 15-hydroxyeicosatetraenoic acid increased in the ARA-administered group, whereas renal concentration of docosahexaenoic acid and eicosapentaenoic acid decreased in the ARA-administered group. Levels of docosahexaenoic acid-derived protectin D1, eicosapentaenoic acid-derived 5-, and 18-hydroxyeicosapentaenoic acids, and resolvin E2 and E3 decreased in the ARA-administered group. Our results indicate that long-term ARA administration led to no serious adverse reactions under normal conditions and to a decrease in anti-inflammatory docosahexaenoic acid- and eicosapentaenoic acid-derived metabolites in the kidneys of aged rats. These results indicate that there is a possibility of ARA administration having a reducing anti-inflammatory effect on the kidney.

## Introduction

Eicosanoids, metabolites derived from arachidonic acid (ARA), have well-established roles in renal physiological and pathophysiological functions [1]. Prostaglandin (PG) I_2_ and PGE_2_ play critical roles in maintaining blood pressure, renal function in a volume-contracted state, and renin secretion [2]. The physiological effects of each eicosanoid are controlled at synthesis and by interactions with its receptors. Therefore, nonsteroidal anti-inflammatory drugs cause fluid and electrolyte disorders, acute renal dysfunction, nephrotic syndrome/interstitial nephritis, and renal papillary necrosis [3–5]. These reports indicate that eicosanoids are important mediators formaintaining renal function.

In contrast, inflammatory cytokines and reactive oxygen species (ROS) activate ARA release from cell membrane phospholipids of the kidney. Huang *et al*. reported that interleukin (IL)-1 rapidly stimulates the release of phospholipase A_2_ (PLA_2_) activity-dependent ARA and activates mesangial cells via the Jun N-terminal/stress-activated protein kinase (JNK/SAPK) signaling pathway [6]. ROS activates renal mitochondrial PLA_2_ activity and cyclooxygenase-2 (COX-2) expression in the kidney [7,8]. The effects of tumor necrosis factor-α (TNF-α) on ion transport are related to the induction of COX-2-dependent PGE_2_ synthesis [9]. These results indicate that endogenous ARA released by inflammatory cytokines and ROS are involved in inflammatory processes in the kidney. Few data regarding whether exogenous ARA stimulates the release of inflammatory cytokines are available. Exogenous ARA but not eicosanoids increases IL-1-dependent ARA release by human embryonic kidney 293 cells via cPLA_2_ and sPLA_2_ [10]; moreover, ARA and its precursor, linoleic acid (LA), directly stimulates the JNK/SAPK pathway [6]. However, the effects of exogenous ARA on kidney function *in vivo* have not been reported because of difficulties in obtaining large quantities of purified ARA. We have assessed whether long-term administration of ARA could change normal renal function, inflammatory, and oxidative state in aged rats. It has been reported that aging is associated with structural and functional renal changes [11,12]. Inflammation has been reported to be a cause of reduced renal function with age [13,14]. Since ARA is known to be involved in inflammation processes as described above, the present study aimed to investigate whether ARA administration decreases kidney function in the aged rats via inflammation. Excess amounts of ARA-derived eicosanoids are known to be involved in inflammatory responses; meanwhile, eicosapentaenoic acid (EPA)- and docosahexaenoic acid (DHA)-derived metabolites have anti-inflammatory properties. We expected ARA administration to disrupt the balance among these metabolite profiles in the kidney. To assess this, we measured the levels of renal eicosanoids and docosanoids using liquid chromatography–electrospray ionization tandem mass spectrometry (LC-ESI-MS/MS).

## Materials and Methods

### Ethics Statement

All animal experiments were conducted in strict accordance with procedures outlined in the Guidelines for Animal Experimentation of Shimane University, compiled from the Guidelines for Animal Experimentation of the Japanese Association for Laboratory Animal Science. The protocol was approved by the Committee on the Ethics of Animal Experiments of the Shimane University.

### Animals and treatments

Rats (Jcl: Wistar) purchased from CLEA Japan (Osaka, Japan) were housed in a room under controlled temperature (23 ± 2°C), humidity (50 ± 10%), and light–dark cycle (light: 07:00–19:00; dark: 19:00–07:00). They were fed a fish oil-deficient diet (F-1™ Funabashi Farm, Funabashi, Japan) and water ad libitum. Inbred second-generation male rats, fed the same F-1 diet, were used for our study. Fatty acid composition in the F-1 diet was described previously [15]. ARA oil was obtained from Cargill Alking Bioengineering (Wuhan and Hubei, China) [16,17]. The fatty acid compositions of the ARA and control oils are shown in Table 1. Twenty one-month-old male rats were divided into two groups: the control group was orally administrated with the control oil and the ARA group was administrated ARA oil (240 mg ARA/kg body weight/day) for 13 weeks. After a 16-h fast, rats were anesthetized with intraperitoneal sodium pentobarbital (65 mg/kg body weight), following which their plasma, kidneys, and liver were removed, immediately frozen in liquid nitrogen, and stored at −30°C until further use. The kidneys and liver were homogenized in phosphate buffer (pH, 7.4) using a Teflon homogenizer (AGC techno glass Co., Ltd. Shizuoka, Japan). The homogenates were immediately frozen in liquid nitrogen and stored at −30°C until use. Concentrations of creatinine and blood urea nitrogen (BUN) were determined in plasma samples using an automatic analyzer (BiOLiS 24i; Tokyo Boeki Medical System Ltd., Tokyo, Japan).

**Table 1:** Fatty acids profile in control and ARA oil.

(%mol)	Control	ARA
PLA (16:0)	13.8 ± 0.01	6.95 ± 0.00
STA (18:0)	13.8 ± 0.01	5.91 ± 0.00
OLA (18:1n-9)	42.5 ± 0.03	5.31 ± 0.00
LA (18:2n-6)	20.0 ± 0.02	9.38 ± 0.01
ALA (18:3n-3)	ND	ND
ARA (20:4n-6)	ND	45.1 ± 0.04
EPA (20:5n-3)	0.13 ± 0.01	0.52 ± 0.00
DPA (22:5n-3)	ND	ND
DHA (22:6n-3)	ND	ND

PLA, palmitic acid; STA, stearic acid, OLA, oleic acid; LA, linolenic acid; ALA, α-Linolenic acid; ARA, arachidonic acid; EPA, eicosapentaenoic acid; DPA, docosapentaenoic acid; DHA, docosahexaenoic acid; ND, not detected.

### Analysis of fatty acid profiles

The fatty acid profiles of the plasma, and kidney, and liver homogenates were determined by gas chromatography, as described previously [18].

### ROS and lipid peroxidation (LPO) measurement

ROS levels were measured as previously described previously [19]. Data are expressed as dichlorofluorescein production/min/mg protein. LPO levels were measured using the thiobarbituric acid reactive substance assay, as described previously [20], and data are expressed as moles of malondialdehyde/mg protein. Protein concentration was determined by the Lowry method [21].

### Sample preparation for analysis of fatty acid metabolites

Kidney homogenates were adjusted to 67% methanol and kept at −30°C, and samples were centrifuged at 5,000 × *g* for 10 min at 4°C to remove precipitated proteins. The supernatants were diluted with ice-cold distilled water and adjusted to 10% (v/v) methanol. Internal standards (5 ng of PGE_2_-*d4*, PGD_2_-*d4*, PGF_2α_-*d4*, and 5-HETE-*d8*, ARA-*d8*) were added to each sample. Samples were acidified to pH 4.0 with 0.1 M HCl and were immediately applied to preconditioned solid-phase extraction cartridges (Sep-Pak C18, Waters, Milford, MA, USA) to extract the fatty acid metabolites. Sep-Pak cartridges were washed with 20 mL water and 20 mL n-hexane in succession. Finally, fatty acid metabolites were eluted with 10 mL methyl formate.

### LC-ESI-MS–MS-based analysis

Fatty acid metabolites in kidneys were measured, as described previously, with a slight modification [22–24]. High-performance liquid chromatography (HPLC) was combined with ESI–MS using a TSQ quantum mass spectrometer (Thermo Fisher Scientific K.K., Tokyo, Japan). HPLC was performed using a Luna 3u C18(2) 100Å LC column (100 × 2.0 mm, Phenomenex, Torrance, CA, USA) at 30°C. Samples were eluted in a mobile phase comprising acetonitrile–methanol (4:1, v/v) and water–acetic acid (100:0.1, v/v) in a 27:73 ratio for 5 min, ramped up to a 70:30 ratio after 15 min, to a 80:20 ratio after 25 min, held for 8 min, ramped up to 100:0 ratio after 35 min, and held for 10 min with flow rate of 0.1 mL/min. MS–MS analyses were conducted in negative ion mode, and fatty acid metabolites were detected and quantified by selected reaction monitoring (SRM). Conditions for the detection of each compound by SRM are listed (S1 Table). Peaks were selected and their areas were calculated using the Xcalibur 2.1 software (Thermo Fisher Scientific K.K.).

### Determination of cytokine levels

Plasma concentrations of IL-1β, IL-4, IL-6, IL10, IL-13, and TNF-α were measured using the Bio-Plex system which combines the principle of a sandwich immunoassay with Luminex fluorescent bead-based technology (Bio-Rad).

### Statistical analysis

Results are expressed as means ± standard errors. Data were analyzed with Student’s t-test. Differences between the groups were considered significant at *P* < 0.05. All statistical analyses were performed using PASW Statistics 18.0 (IBM-SPSS, Inc., Armonk, NY, USA).

## Results

### Renal function parameters and plasma cytokine levels

**Fig 1:**
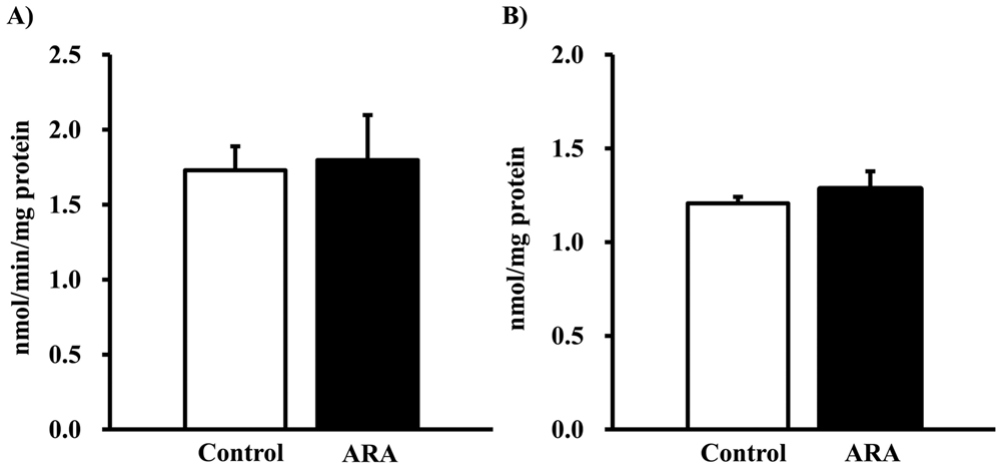
Effects of arachidonic acid administration on renal levels of reactive oxygen species and lipid peroxidation. (A) Reactive oxygen species and (B) lipid peroxide levels in the kidney. Values are expressed as means ± standard error (n = 14–16) percentages relative to the control. * *P* < 0.05 versus control group.

A comparison of levels of plasma creatinine, BUN, and cytokine is summarized in Table 2. Levels of plasma creatinine and BUN were not significantly different between the two groups. No significant differences were observed between the two groups for plasma levels of inflammatory and anti-inflammatory cytokines. ROS and LPO levels in the kidney were not significantly different between the two groups (Fig 1).

**Table 2:** Biochemical data and cytokine levels in plasma of ARA treated aged rats.

	Control group	ARA group
Creatine (mg/dL)	0.30 ± 0.02	0.33 ± 0.01
Blood urea nitrogen (mg/dL)	17.2 ± 0.6	18.4 ± 0.6
IL-1β (ng/mL)	99.8 ± 11.3	82.1 ± 14.1
IL-4 (ng/mL)	7.78 ± 0.49	7.50 ± 0.73
IL-6 (ng/mL)	37.9 ± 9.1	35.2 ± 18.5
IL-10 (ng/mL)	84.8 ± 13.6	126.7 ± 13.7
IL-13 (ng/mL)	16.1 ± 2.0	14.0 ± 3.7
TNF-α (ng/mL)	15.5 ± 4.6	11.9 ± 4.4

IL, Interleukin; TNF, tumor necrosis factor

Values are means ± SEM for 14–16 rats. There were not statistically significant differences between groups.

### Plasma, kidney, and liver fatty acid profiles

Fatty acid profiles in the plasma after 13 weeks of administration are shown in Table 3. ARA concentration increased significantly in the ARA-administered group, whereas eicosapentaenoic acid (EPA) concentration in plasma decreased significantly in the ARA-administered group. The docosahexaenoic acid (DHA)/ARA and EPA/ARA ratios decreased significantly, whereas the n-6/n-3 ratio increased in the ARA-administered group. Mole percentages of oleic acid, linoleic acid (LA), and DHA decreased significantly in the ARA-administered group (data not shown). Fatty acid profiles in the kidney after 13 weeks of administration are shown in Table 4. ARA concentration increased significantly, whereas EPA and DHA concentrations decreased significantly in the ARA-administered group. The DHA/ARA and EPA/ARA ratios decreased significantly, whereas the n-6/n-3 ratio increased in the ARA-administered group, which were similar to those in plasma. Fatty acid profiles in the liver after 13 weeks of administration are shown (S2 Table). EPA and OLA concentrations in the liver decreased significantly in the ARA-administered group, whereas ARA concentration increased significantly in the ARA-administered group. The DHA/ARA and EPA/ARA ratios decreased significantly, whereas the n-6/n-3 ratio increased in the ARA-administered group.

**Table 3:** Effects of chronic ARA treatment on fatty acid profiles in plasma of aged rats.

	Control group	ARA group
PLA (16:0) (μg/mL)	652.76 ± 43.03	677.29 ± 35.21
STA (18:0) (μg/mL)	400.59 ± 47.01	392.75 ± 13.97
OLA (18:1n-9) (μg/mL)	322.44 ± 36.52	301.09 ± 22.64
LA (18:2n-6) (μg/mL)	550.68 ± 46.13	526.90 ± 45.94
ALA (18:3n-3) (μg/mL)	9.95 ± 1.42	9.61 ± 1.27
ARA (20:4n-6) (μg/mL)	795.43 ± 70.56	1031.84 ± 45.56*
EPA (20:5n-3) (μg/mL)	10.95 ± 0.84	7.11 ± 0.95*
DPA (22:5n-3) (μg/mL)	13.54 ± 0.98	13.88 ± 0.86
DHA (22:6n-3) (μg/mL)	66.25 ± 12.63	53.68 ± 5.00
n-6/n-3 ratio (mol/mol)	16.07 ± 1.25	20.92 ± 0.90*
DHA/ARA ratio (mol/mol)	0.074 ± 0.007	0.047 ± 0.003*
EPA/ARA ratio (mol/mol)	0.016 ± 0.002	0.007 ± 0.001*

PLA, palmitic acid; STA, stearic acid, OLA, oleic acid; LA, linolenic acid; ALA, α-Linolenic acid; ARA, arachidonic acid; EPA, eicosapentaenoic acid; DPA, docosapentaenoic acid; DHA, docosahexaenoic acid; n-6, n-6 polyunsaturated fatty acids; n-3, n-3 polyunsaturated fatty acids.

Values are means ± SEM for 14–16 rats.

* Significantly different from control group (*P* < 0.05).

**Table 4:** Effects of chronic ARA treatment on fatty acid profiles in kidney of aged rats.

	Control group	ARA group
PLA (16:0) (μg/mg protein)	51.97 ± 4.36	54.87 ± 3.89
STA (18:0) (μg/mg protein)	32.26 ± 1.31	33.62 ± 0.80
OLA (18:1n-9) (μg/mg protein)	37.39 ± 5.49	39.89 ± 5.35
LA (18:2n-6) (μg/mg protein)	36.39 ± 4.02	38.08 ± 4.95
ALA (18:3n-3) (μg/mg protein)	0.75 ± 0.14	0.80 ± 0.14
ARA (20:4n-6) (μg/mg protein)	37.93 ± 1.53	44.50 ± 0.78*
EPA (20:5n-3) (μg/mg protein)	0.31 ± 0.02	0.17 ± 0.01*
DPA (22:5n-3) (μg/mg protein)	0.51 ± 0.04	0.47 ± 0.02
DHA (22:6n-3) (μg/mg protein)	2.75 ± 0.20	2.29 ± 0.09*
n-6/n-3 ratio (mol/mol)	18.98 ± 0.76	23.83 ± 0.53*
DHA/ARA ratio (mol/mol)	0.067 ± 0.004	0.048 ± 0.001*
EPA/ARA ratio (mol/mol)	0.008 ± 0.001	0.004 ± 0.001*

PLA, palmitic acid; STA, stearic acid, OLA, oleic acid; LA, linolenic acid; ALA, α-Linolenic acid; ARA, arachidonic acid; EPA, eicosapentaenoic acid; DPA, docosapentaenoic acid; DHA, docosahexaenoic acid; n-6, n-6 polyunsaturated fatty acids; n-3, n-3 polyunsaturated fatty acids.

Values are means ± SEM for 14–16 rats.

* Significantly different from control group (*P* < 0.05).

### Levels of eicosanoids and docosanoids in the kidney

**Fig 2:**
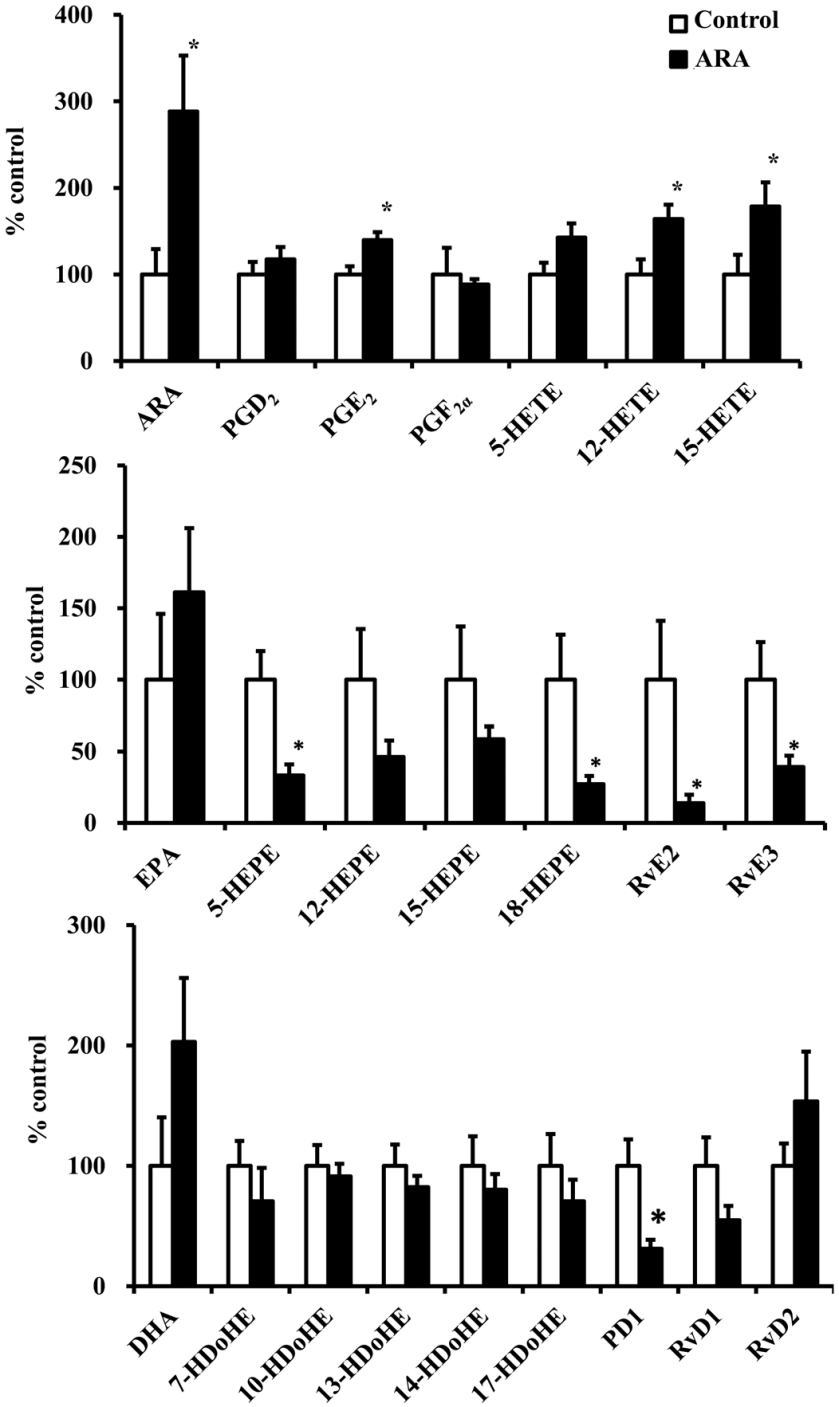
Renal levels of arachidonic acid (ARA)-, eicosapentaenoic acid (EPA)-, and docosahexaenoic acid (DHA)-derived metabolites. Kidney samples were subjected to liquid chromatography–tandem mass spectrometry (LC-MS/MS). (A) ARA-, (B) EPA-, and (C) DHA-derived metabolites. Values are expressed as means ± standard error (n = 14–16) percentages relative to the control. * *P* < 0.05 versus control group.

Kidney analyses revealed an increase in renal formation of PGE_2_, 12-HETE, and 15-HETE in the ARA-administered group (Fig 2). Moreover, endogenous formation of DHA-derived PD1 and EPA-derived 5-HEPE, 18-HEPE, RvE2, and RvE3 decreased significantly in the ARA-administered group. Nonesterified ARA levels in the kidney increased significantly in the ARA-administered group, whereas levels of nonesterified EPA and DHA were not significantly different between the two groups.

## Discussion

One of the purposes of this study was to assess the effects of long-term ARA administration on kidney function. Plasma levels of BUN and creatinine are usually used in conjunction to measure kidney function, help diagnose kidney disease, and monitor kidney status. Long-term ARA administration did not affect levels of BUN and creatinine, suggesting that ARA-administration did not decrease renal function in aged rats. Next we assessed the levels of LPO and ROS as well as inflammatory cytokine levels in plasma; these were not affected by the ARA treatment, suggesting that ARA-administration itself did not cause inflammation and oxidative stress. These results agree with those of our previous study [16]. Yoshizawa et al. reported that an ARA-rich diet for dams during gestation and lactation does not modify *N*-methyl-*N*-nitrosourea-induced renal preneoplastic lesions in their offspring [25], indicating that exogenous free-form ARA does not induce inflammation or oxidative stress in the kidney.

The ARA concentration increased significantly in plasma, kidney, and liver after long-term ARA administration (Tables 3 and 4; S2 Table), and the calculated liver-to-plasma concentration ratio was 6.57 ± 0.44 in the control group and 6.51 ± 0.28 in the ARA group (*P* = 0.902); moreover, the kidney-to-plasma concentration ratio in the control and ARA group was 5.15 ± 0.35 and 4.43 ± 0.19, respectively (*P* = 0.085), indicating that orally administered ARA was well absorbed from the intestinal tract and was distributed in the kidney and liver; also, long-term ARA administration did not change the distribution from plasma to the liver and kidneys. These results agree with those of previous studies. Our previous study showed that oral ARA administration for 13 weeks significantly increased ARA levels in plasma [16]. Zhou *et al*. reported that free-form ARA is distributed from plasma to several tissues. The retention rate of ARA in the heart, lungs, kidneys, and bone marrow is higher than that in other tissues, but lower than that in the liver [26].

This is the first report to measure eicosanoids and docosanoids levels in the kidney of ARA administrated rats. It is well known that linoleic acid (LA) but not ARA administration increases ARA-derived metabolites in the kidneys [27]. It has also been reported that cytochrome P450-derived ARA metabolites are increased in kidney microsomes incubated with ARA *in vitro* [28]; no study has been published, in which ARA-derived metabolites of the kidneys were quantified *in vivo* following ARA administration. Chronic ARA administration increased levels of PGE_2_, 12-HETE, and 15-HETE in the kidney. PGE_2_ treatment promotes resolution of glomerular inflammation [29]. 15-HETE is capable of antagonizing the pro-inflammatory actions of leukotriene B_4_ in the rat [30]. 15-HETE antagonizes leukotriene-induced neutrophil chemotaxis in glomerular microcirculation [31]. It has been reported that 12-HETE is synthesized by renal cortical tissue and reduces basal renin release [32,33]. A previous study reported that these eicosanoids consistently fail to enhance IL-1-stimulated JNK1/SAPK activity [6]. These results indicate that increasing these eicosanoids by chronic ARA administration does not stimulate inflammation in the healthy kidney; on the contrary, the concentration of eicosanoids with renoprotective properties increased.

In contrast to healthy kidneys, long-term treatment with omega-6 PUFA causes severe inflammatory response in a rat renal ischemia-reperfusion injury model. COX-2 and LOX are induced during rat renal ischemia-reperfusion injury [34]. Long-term treatment with omega-6 PUFA LA, a precursor of ARA, significantly elevates serum creatinine levels as a result of 30 min of renal ischemiaand extends ischemia to 45 min caused 100% mortality in the omega-6 PUFA group, in contrast to 0% mortality in the omega-3 PUFA group [27]. Indomethacin (COX inhibitor)-treated mice present with better renal function and less acute tubular necrosis, reduced ROS, and lower expression of pro-inflammatory cytokines during acute kidney injury [35]. Taken together, these results indicate that increasing ARA levels as well as its metabolites in the injured kidneys may result in severe kidney damage. Because the administrated free-form ARA was taken up by the kidneys and was acylated into phospholipids for the plasma membrane and cell nuclei [26,36], long-term ARA administration did not induce inflammation or oxidative stress in the present study; however, eicosanoid production in ARA administered rats may markedly increase tissue injury and inflammation because of robust activation of phospholipases and downstream biosynthetic pathways. It has been reported that eicosanoids exert diverse and complex functions. In addition to their role in regulating normal kidney function, these lipids also play important roles in the pathogenesis of kidney diseases [37]. The present study also demonstrated that ARA-derived eicosanoids do not induce renal inflammation and oxidative stress in aged rats.

Concentrations of non-esterified EPA and DHA increased slightly, but not significantly, in the ARA-administered group compared with those in the control group, although total EPA and DHA concentrations decreased in the kidney. Levels of EPA-derived eicosanoids 5-HEPE, 18-HEPE, RvE2, and RvE3 and the DHA-derived docosanoids PD1 decreased significantly in the ARA-administered group. These results suggest that ARA directly competes with the storage of EPA and DHA at the sn-2 position in phospholipids and blocks the production of EPA- and DHA-derived metabolites [38]. We have reported that the DHA-derived docosanoids RvDs and PD1 protect renal damage progression induced by metabolic syndrome [24]. EPA-derived eicosanoids and DHA-derived docosanoids are endogenous mediators with potent anti-inflammatory actions in the kidneys [39]. Administration of RvDs or PD1 to mice prior to ischemia results in a reduction in functional and morphological renal injury [40]. Hong and Lu demonstrated that RvDs and PD1 repress renal interstitial fibrosis, and PD1 inhibits the inflammatory response and promotes renoprotective heme-oxygenase-1 expression during acute kidney injury [41]. Heme-oxygenase-1 decreases acute tubular necrosis and significantly reduces COX-2 and microsomal PGE synthase expression [42]. Our study demonstrated that RvE2, RvE3, and PD1 levels decreased significantly in the ARA-administered group, indicating that anti-inflammatory defense may be attenuated by ARA administration.

In conclusion, ARA-derived eicosanoids are important regulators that maintain physiological renal functions. In addition, ARA-derived eicosanoids enhance the pathological response to renal injury. We assessed the impact of long-term ARA administration on normal renal function as well as on inflammation and oxidative stress. Our results demonstrate that ARA levels in the plasma, kidneys, and liver increased following ARA treatment. In addition, levels of PGE_2_, 12-HETE, and 15-HETE increased, and those of DHA-derived PD1, EPA-derived 5-HEPE, 18-HEPE, and RvE3 decreased in the ARA-administered group. However, kidney function, levels of inflammatory cytokines, and oxidative stress were not affected by ARA treatment. Taken together, our results indicate that long-term ARA administration has no serious adverse effects under normal condition; however, further studies are needed to assess the risk of long-term ARA treatment in animal models of kidney injury.

## Supporting Information

S1 Table Selected reaction monitoring (SRM) transitions of fatty acid metabolites. PG, prostaglandin; HETE, hydroxyeicosatetraenoic acid; HEPE, hydroxyeicosapentaenoic acids; Rv, Resolvin; HDoHE, hydroxydocosahexaenoic acid; PD1, Protectin D.
(DOCX)

S2 Table Effects of chronic ARA treatment on fatty acid profiles in liver of aged rats. PLA, palmitic acid; STA, stearic acid, OLA, oleic acid; LA, linolenic acid; ALA, α-Linolenic acid; ARA, arachidonic acid; EPA, eicosapentaenoic acid; DPA, docosapentaenoic acid; DHA, docosahexaenoic acid; n-6, n-6 polyunsaturated fatty acids; n-3, n-3 polyunsaturated fatty acids. Values are means ± SEM for 14–16 rats. * Significantly different from control group (*P* < 0.05).
(DOCX)
