# Supplementary material for: Chronic Arachidonic Acid Administration Decreases Docosahexaenoic Acid- and Eicosapentaenoic Acid-Derived Metabolites in Kidneys of Aged Rats
Source: PLoS One. 2015 Oct 20;10(10):e0140884. doi: 10.1371/journal.pone.0140884 (PMC4618288; doi:10.1371/journal.pone.0140884)
Supplement: S1 Table — PG, prostaglandin; HETE, hydroxyeicosatetraenoic acid; HEPE, hydroxyeicosapentaenoic acids; Rv, Resolvin; HDoHE, hydroxydocosahexaenoic acid; PD1, Protectin D. (DOCX) [file pone.0140884.s001.docx]

| Compound | SRM Transition  (m/z) | Compound | SRM Transition  (m/z) | Compound | SRM Transition  (m/z) |
| --- | --- | --- | --- | --- | --- |
| ARA | 303>259 | EPA | 301>257 | DHA | 327>283 |
| PGE_2_ | 351>271 | 5-HEPE | 317>115 | 7-HDoHE | 343>141 |
| PGD_2_ | 351>271 | 12-HEPE | 317>179 | 10-HDoHE | 343>153 |
| PGF_2α_ | 353>193 | 15-HEPE | 317>219 | 13-HDoHE | 343>193 |
| 5-HETE | 319>115 | 18-HEPE | 317>259 | 17-HDoHE | 343>245 |
| 12-HETE | 319>179 | RvE2 | 333>115 | PD1 | 359>153 |
| 15-HETE | 319>219 | RvE3 | 333>213 | RvD1 | 375>141 |
| AA-*d*_8_ | 311>267 |  |  | RvD2 | 375>175 |
| PGE_2_-*d*_4_ | 355>275 |  |  |  |  |
| PGD_2_-*d*_4_ | 355>275 |  |  |  |  |
| PGF_2α_-*d*_4_ | 357>197 |  |  |  |  |
| 5-HETE-*d*_8_ | 327>116 |  |  |  |  |

**Supporting Information Captions**


**S1 Table 1. Selected reaction monitoring (SRM) transitions of fatty acid metabolites.** PG, prostaglandin; HETE, hydroxyeicosatetraenoic acid; HEPE, hydroxyeicosapentaenoic acids; Rv, Resolvin; HDoHE, hydroxydocosahexaenoic acid; PD1, Protectin D.
