# Supplementary material for: Chronic Arachidonic Acid Administration Decreases Docosahexaenoic Acid- and Eicosapentaenoic Acid-Derived Metabolites in Kidneys of Aged Rats
Source: PLoS One. 2015 Oct 20;10(10):e0140884. doi: 10.1371/journal.pone.0140884 (PMC4618288; doi:10.1371/journal.pone.0140884)
Supplement: S2 Table — PLA, palmitic acid; STA, stearic acid, OLA, oleic acid; LA, linolenic acid; ALA, α-Linolenic acid; ARA, arachidonic acid; EPA, eicosapentaenoic acid; DPA, docosapentaenoic acid; DHA, docosahexaenoic acid; n-6, n-6 polyunsaturated fatty acids; n-3, n-3 polyunsaturated fatty acids. Values are means ± SEM for 14–16 rats. * Significantly different from control group (P < 0.05). (DOCX) [file pone.0140884.s002.docx]

|  | Control group | ARA group |
| --- | --- | --- |
| PLA (16:0) (μg/mg protein) | 68.87 ± 2.35 | 65.02 ± 1.61 |
| STA (18:0) (μg/mg protein) | 57.48 ± 1.58 | 59.68 ± 0.88 |
| OLA (18:1n-9) (μg/mg protein) | 29.19 ± 1.79 | 21.88 ± 1.15* |
| LA (18:2n-6) (μg/mg protein) | 50.14 ± 4.17 | 41.79 ± 3.09 |
| ALA (18:3n-3) (μg/mg protein) | 0.92 ± 0.12 | 0.68 ± 0.07 |
| ARA (20:4n-6) (μg/mg protein) | 48.56 ± 2.06 | 65.59 ± 1.53* |
| EPA (20:5n-3) (μg/mg protein) | 0.60 ± 0.04 | 0.28 ± 0.02* |
| DPA (22:5n-3) (μg/mg protein) | 1.99 ± 0.11 | 2.07 ± 0.05 |
| DHA (22:6n-3) (μg/mg protein) | 11.06 ± 0.67 | 9.59 ± 0.68 |
|  |  |  |
| n-6/n-3 ratio (mol/mol) | 7.55 ± 0.32 | 9.59 ± 0.33* |
| DHA/ARA ratio (mol/mol) | 0.21 ± 0.01 | 0.13 ± 0.01* |
| EPA/ARA ratio (mol/mol) | 0.013 ± 0.001 | 0.004 ± 0.0002* |

**Supporting Information Captions**

**S2 Table. Effects of chronic ARA treatment on fatty acid profiles in liver of aged rats.** PLA, palmitic acid; STA, stearic acid, OLA, oleic acid; LA, linolenic acid; ALA, α-Linolenic acid; ARA, arachidonic acid; EPA, eicosapentaenoic acid; DPA, docosapentaenoic acid; DHA, docosahexaenoic acid; n-6, n-6 polyunsaturated fatty acids; n-3, n-3 polyunsaturated fatty acids. Values are means ± SEM for 14–16 rats. * Significantly different from control group (*P* < 0.05).
